# Targeted genome modification in protoplasts of a tea cultivar Kolkhida using RNA-guided Cas9 endonuclease

**DOI:** 10.1093/aobpla/plaf056

**Published:** 2025-10-04

**Authors:** Anastasiya Egorova, Ivan Fomin, Anastasia Fizikova, Nina Kostina, Lyudmila Malyukova, Lidiia Samarina, Sophia Gerasimova

**Affiliations:** Laboratory of Molecular and Genetic Research, Federal Research Centre the Subtropical Scientific Centre of the Russian Academy of Sciences, Yana Fabritsiusa str. 2/28, Sochi, Krasnodar Krai 354002, Russia; Laboratory of Gene Engineering, Federal Research Centre the Institute of Cytology and Genetics of the Siberian Branch of the Russian Academy of Sciences, Ac. Lavrentieva ave. 10, Novosibirsk 630090, Russia; Laboratory of Gene Engineering, Federal Research Centre the Institute of Cytology and Genetics of the Siberian Branch of the Russian Academy of Sciences, Ac. Lavrentieva ave. 10, Novosibirsk 630090, Russia; Laboratory of Molecular and Genetic Research, Federal Research Centre the Subtropical Scientific Centre of the Russian Academy of Sciences, Yana Fabritsiusa str. 2/28, Sochi, Krasnodar Krai 354002, Russia; Center of Genetics and Life Sciences, Sirius University of Science and Technology, Olympiyskiy ave. 1, Sirius Federal Territory, Krasnodar Krai 354340, Russia; Laboratory of Gene Engineering, Federal Research Centre the Institute of Cytology and Genetics of the Siberian Branch of the Russian Academy of Sciences, Ac. Lavrentieva ave. 10, Novosibirsk 630090, Russia; Laboratory of Molecular and Genetic Research, Federal Research Centre the Subtropical Scientific Centre of the Russian Academy of Sciences, Yana Fabritsiusa str. 2/28, Sochi, Krasnodar Krai 354002, Russia; Laboratory of Molecular and Genetic Research, Federal Research Centre the Subtropical Scientific Centre of the Russian Academy of Sciences, Yana Fabritsiusa str. 2/28, Sochi, Krasnodar Krai 354002, Russia; Center of Genetics and Life Sciences, Sirius University of Science and Technology, Olympiyskiy ave. 1, Sirius Federal Territory, Krasnodar Krai 354340, Russia; Laboratory of Molecular and Genetic Research, Federal Research Centre the Subtropical Scientific Centre of the Russian Academy of Sciences, Yana Fabritsiusa str. 2/28, Sochi, Krasnodar Krai 354002, Russia; Laboratory of Gene Engineering, Federal Research Centre the Institute of Cytology and Genetics of the Siberian Branch of the Russian Academy of Sciences, Ac. Lavrentieva ave. 10, Novosibirsk 630090, Russia

**Keywords:** *Camellia sinensis*, Cas9/gRNA, protoplasts, gene editing, genetic transformation

## Abstract

Gene-editing tools enable precise, targeted genome modifications, providing new approach for the rapid and sustainable improvement of tea plant (*Camellia sinensis* (L.) Kuntze). Developing such an approach is especially important due to the perennial nature and complex genetics of the tea plant, which make traditional breeding slow and inefficient. To validate a gene editing protocol in the elite local tea cultivar Kolkhida three candidate genes were selected. Two guide RNAs (gRNAs) were designed for each gene, and corresponding constructs for targeted genome modification in tea were generated. Successful modifications of the target sequences in cv. Kolkhida tea protoplasts were achieved for all three target genes. The high mutagenic efficiency of the selected gRNAs was observed for two out of three genes, including induction of precise deletions between target motifs. gRNAs were delivered in protoplasts via co-transfection technique, and combined gRNA activity was observed when transfection efficiency exceeded 28%. The genome modification method for tea protoplasts established in this study can serve as a screening protocol to evaluate the *in vivo* efficiency of different genome editing approaches in the tea plant.

## Teaser

The genome modification method for tea protoplasts was established. Three candidate genes were selected in the elite local tea cultivar Kolkhida. Constructs for targeted genome modification were generated using two gRNAs per gene. High mutagenic efficiency of the selected gRNAs was observed for two out of three genes, including the induction of precise deletions between target motifs. Combined gRNA activity was detected when transfection efficiency exceeded 28%.

## Introduction

Tea (*Camellia sinensis* (L.) Kuntze) is a perennial woody plant and one of the most important agricultural crops for beverage, medicinal, and functional applications. It is cultivated in over fifty countries worldwide (Chen et al. 2012). Tea improvement through conventional breeding remains challenging due to the plant's long generation time, self-incompatibility, and high heterozygosity (Chen et al. 2012). Key agronomic challenges such as stress tolerance, disease resistance, and leaf quality traits of tea plant can be addressed by application of biotechnological approaches instead of classical breeding. However, significant bottlenecks remain in the development of effective methods for both functional genomics and biotechnology (Mukhopadhyay et al. 2016). Particularly, stable genetic *Agrobacterium*-mediated transformation is time- and labour-consuming, and still inefficient (Jin et al. 2022). Tea explants require approximately 8–12 months of selection and regeneration. Metabolites released by tea affect explant viability, making the transformation and regeneration difficult (Mohanpuria et al. 2011, Fizikova et al. 2024).

RNA-guided Cas9 endonuclease-based gene editing has been shown to be efficient for wide-ranging applications in crop improvement (Koeppel et al. 2019, Korotkova et al. 2019). One of the main challenges for agricultural scientists is to integrate this method into plant breeding and biotechnology for rapid development of new crop varieties with desired traits, especially with enhanced tolerance to environmental stresses. Reliable genetic transformation and genome editing protocols have been published for several woody plants, such as *Populus* (poplar) and *Malus domestica* (apple), providing valuable models for gene functional analysis and precise breeding (Min et al. 2022). These advances underscore the potential for similar progress in tea plant biotechnology however, the appropriate model systems and protocols for tea genome editing are still to be established.

Previously, Ma et al. (2021) successfully introduced targeted mutations in the *CsHB1* gene by transforming tea callus using the gRNA/Cas9 gene editing system, however stable, whole-grown mutant tea plants have not yet been obtained. The establishment of an efficient and reproducible gene editing platform in tea protoplasts would be a critical step towards the development of genome editing approaches in tea. Transient expression in protoplasts has been widely used for optimization of genome editing systems, including genetic construct designs, and assessment the efficiency of different Cas proteins and guide RNAs (gRNAs) (Brandt et al. 2020, Badhan et al. 2021). In our previous studies, we successfully employed protoplast-based pre-evaluation and optimization of Cas9/gRNA constructs in potato, barley, and maize (Gerasimova et al. 2018, Hoffie et al. 2021, Hernandes-Lopes et al. 2023, Egorova et al. 2025, 2024). While several protocols have been established for the isolation and transfection of tea protoplasts (Xu et al. 2021, Zhou et al. 2021, Kumar et al. 2023), genome editing in tea protoplasts has not yet been reported. Thus, the aim of this study was the development of a protocol for targeted genome modifications in tea protoplasts. We successfully performed targeted modification of the candidate genes potentially involved in cold adaptation of tea plants. Developed protocol can be used for any further genome editing applications in tea.

## Materials and methods

### Plant material and protoplast isolation

The North Caucasus tea plant (*Camellia sinensis* var. *sinensis*) cultivar Kolkhida was used as the donor material for protoplast isolation. Among local cultivars, Kolkhida is considered one of the best cultivar for black and green tea production (Daraselia et al. 1989).

For protoplast isolation, both *in vivo* and *in vitro* plants were used. The *in vivo* plants were one-year-old seedlings that were grown in pots filled with acid peat (pH ~ 5) (Terra Vita, Russia). They were grown from April to October, at room temperature under natural light. *In vitro* plants were grown on MS culture medium (M0222, Duchefa, The Netherlands) (Murashige and Skoog 1962) supplemented with 2% sucrose, 0.7% agar (P1001, Duchefa), 1 mg/L NAA (naphthalene-1-acetic acid) (N0640, Sigma-Aldrich, US) and 2 mg/L IBA (indole-3-butyric acid) (I5386, Sigma-Aldrich).

Mesophyll protoplasts were isolated based on a protocol developed earlier (Egorova et al. 2020) with modifications described below. Three to four young, tender upper leaves were used for protoplast isolation. Leaves were cut into strips (0.5–1.0 mm) using sharp scissors and placed in a 15 ml enzymatic solution [20 mM MES (M3621, Sigma-Aldrich), 0.6 M mannitol (M0803, Duchefa), 20 mM KCl (P0515, Duchefa), 10 mM CaCl2 (C4433, Chimmed, Russia), 250 mg/l PVP (PVP10, Sigma-Aldrich), 0.1% bovine serum albumin (05470, Sigma-Aldrich), 1.5% cellulase R10 (C8001, Duchefa), 0.4% Macerozyme R10 (M8002, Duchefa); pH 5.7]. The tissue was incubated in the dark at 20°C with gentle shaking at 20 rpm overnight. Then, 35 ml of the WS solution (2 mM MES, 154 mM NaCl (1.06404, Chimmed, Russia), 5 mM KCl and 125 mM CaCl₂ at pH 5.7) was added, with the following filtration through a 40 µm cell strainer (Corning™) and centrifugation at 100 g for 10 minutes. The upper phase was carefully discarded without disturbing the pelleted protoplasts. The protoplasts were then resuspended in TB solution (0.1% MES, 0.5 M mannitol, and 15 mM MgCl₂ (141396, AppliChem, Germany) at pH 5.7) by gentle swirling and left on ice for 1 hour. The TB solution was then carefully discarded and replaced with a fresh solution. A haemocytometer was used to determine the protoplast yield.

For transfection, 50 000 protoplasts in 200 µl were mixed with plasmids (5 µg of each in 20 µl of water) and 220 µl of PEG solution (40% PEG 6000 (P0805, Duchefa), 0.5 M mannitol, and 15 mM MgCl₂). The samples were then incubated in the dark for 30 minutes. The transfection reactions were stopped by adding 1 ml of WS solution. The samples were then centrifuged at 50 g for 5 minutes. The supernatant was discarded, and the protoplasts were gently resuspended in 2 ml of WS solution.

### Guide RNA design

Three potential cold tolerance genes were identified based on transcriptional analysis of the cv. Kolkhida and selected for gene editing (Samarina et al. 2023).

Target gene sequences were extracted from cv. Kolkhida genome assembly (https://db.cngb.org/search/project/CNP0005366). The coding sequences of the *COR413PM1-like* (TEAK041213), *CRY1* (TEAK018624) and *ELIP1* (TEAK023638)genes were used to select target motifs as previously described (Gerasimova et al. 2018, Gerasimova et al. 2020). It was shown that application of two gRNAs simultaneously increases efficiency of mutagenesis and induces PCR-detectable deletions in plants (Zhou et al. 2014, Do et al. 2019, Salonia et al. 2022). Therefore, it was decided that two target motifs should be selected for each gene (see Supplementary Table S1), and that the efficiencies of gRNA pairs for inducing point indels and long deletions should be tested.

Guide RNAs with high prediction scores and proper secondary structures were selected according to Koeppel et al. (2019). The selection was performed using the online tool WuCRISPR (Wong et al. 2015). Off-target analysis was performed via BLAST of target sequences at the NCBI (https://blast.ncbi.nlm.nih.gov/Blast.cgi).

To confirm the sequence of target motifs, fragments containing the target motifs of the genes were amplified using KAPA3G Plant PCR Kit (KK7251, Kapa Biosystems, South Africa) and sequenced. Primer pairs COR_F and COR_R, CRY_F and CRY_R, ELIP_F and ELIP_R (Supplementary Table S2) were used to amplify gene fragments. Deep sequencing was performed on the Illumina MiSeq platform in the Shared Facility Centre for Genomic Research at the ICG SBRAS (Novosibirsk, Russia). Alignments to the reference were visualized using ENDscript (https://endscript.ibcp.fr; Robert and Gouet 2014). Sequenced fragments including six target motifs were identical to the reference sequence, with the exception of a six-nucleotide deletion in the CRY1 gene (990–995 bp on the genomic sequence) (Supplementary Figure S3).

### Plasmid construction

Double-stranded oligos were generated by melting and reannealing the following pairs of oligonucleotides: COR-1-F and COR-1-R (for gRNA COR-1) and COR-2-F and COR-2-R (for gRNA COR-2) for the knockout of the *COR413PM1-like* gene; CRY-1-F and CRY-1-R (for gRNA CRY-1) and CRY-2-F and CRY-2-R (for gRNA CRY-2) for the knockout of the *CRY1* gene; ELIP-1-F and ELIP-1-R (for gRNA ELIP-1) and ELIP-2-F and ELIP-2-R (for gRNA ELIP-2) for the knockout of the *ELIP1* gene (Supplementary Table S1). The oligonucleotides were then cloned into the pSI57 vector (Kostina et al. 2020), with the gRNA expression cassette under the control of the *A. thaliana* U6-26 promoter and the *A. thaliana* codon-optimized SpCas9 under the control of the parsley PcUbi4-2 promoter.

The following vectors were generated: pIF20 for gRNA COR-1 expression; pIF21 for gRNA COR-2 expression; pIF24 for gRNA CRY-1 expression; pIF25 for gRNA CRY-2 expression; pIF27 for gRNA ELIP-1 expression; pIF28 for gRNA ELIP-2 expression.

### Protoplast transfection and mutation detection

A mixture of three vectors was used in each transfection reaction. Two gRNA/Cas9 vectors were used to induce mutations in two target motifs within the target gene, alongside with the pNB2 vector (Budhagatapalli et al. 2016), containing mCherry reporter gene. The following vector combinations were used: pIF21 + pIF20 + pNB2 for *COR413PM1-like*; pIF24 + pIF25 + pNB2 for *CRY1*; and pIF27 + pIF28 + pNB2 for *ELIP1* gene respectively. Each transfection was performed in four independent replicates. The transfected protoplasts were then incubated in the dark at room temperature (approximately 23°C) for two days.

After incubation, the percentage of protoplasts expressing mCherry in each sample was determined using a fluorescence microscope (EVOS M5000, Thermo Fisher Scientific, US). Genomic DNA was then isolated by spinning down the protoplasts, vortexing them and incubating them for one hour at 55°C in 20 µl of PBND lysis solution containing 50 mM KCl, 10 mM Tris (Am0497, Helicon, Russia) (pH 8.3), 2.5 mM MgCl₂, 0.1 mg/ml gelatine (G2500, Sigma-Aldrich), 0.45% NP-40 (ASN2737, Avra Synthesis, India), 0.45% Tween 20 (8506, NeoFroxx, Germany), and 100 μg/ml Proteinase K (EK001, Evrogen, Russia). The mixture was then heated at 95°C for 10 minutes.

One microliter of the mixture was used for PCR. The target regions were amplified using the following primers: COR_F and COR_R, CRY_F and CRY_R, ELIP_F and ELIP_R (see Supplementary Table S1). Amplicon sequencing was performed using the Illumina MiSeq platform, generating paired-end reads of 250 bp. After filtration, the sequence depth was around 1000–2000 reads per amplicon sample. Mutation frequencies and patterns were analysed individually for each replicate using the Small_Indel_Analyser (SIA) script (https://github.com/vikhall/Small_Indell_Analyzer), which has been previously validated in maize and potato (Hernandes-Lopes et al. 2023, Egorova et al. 2025). Mutation pattern and frequency were assessed by calculating the proportion of mutated reads relative to the total number of reads, normalized to the proportion of transfected (mCherry-positive) protoplasts. The correlation between transfection and mutagenesis efficiencies was estimated using the Spearman correlation test (performed in R, version 4.3.2).

## Results and discussion

Efficiency and pattern of mutations induced by gRNA/Cas9 system in protoplasts are shown to be similar to those at the whole plant level (Egorova et al. 2024). The pre-evaluation of genetic constructs for targeted genome modification in plant protoplasts is being actively used in crops in order to optimize system before time and labor-consuming step of stable plant transformation and regeneration (Gerasimova et al. 2018, Badhan et al. 2021, Hernandes-Lopes et al. 2023). The protoplast test system for genome editing has not yet been implemented in tea plants. This study closes the gap and offers a reliable protocol for tea protoplast transfection and targeted genome modification.

The type of donor plant material is a critical factor affecting the efficiency of protoplast isolation and the subsequent viability of the cells (Chen et al. 2023). Additionally, various factors, such as tissue type, developmental stage and cultivation conditions, can significantly affect the yield and quality of isolated protoplasts (Li et al. 2022). Although callus is one of the most commonly used materials for protoplast isolation (Rahmani et al. 2016, Bertini et al. 2019, Wang et al. 2022) we were unable to efficiently isolate protoplasts from callus of the tea cv. Kolkhida (data are not shown). It was decided to isolate protoplasts from leaves. In our preliminary experiments, tender leaves and young tissues in tea plants have been found to yield greater numbers of viable protoplasts as compared to mature leaves. This can be due to the greater lignification of the mature leaves, making them more recalcitrant to enzymatic degradation (Xu et al. 2021, Kumar et al. 2023). Two different types of young leaves were used in this study.

In total, between 300 000 and 1 250 000 protoplasts were isolated from 1 g of fresh leaves grown *in vitro* and *in vivo*, respectively. The smaller number of viable protoplasts obtained from *in vitro*-grown leaves can be explained by the greater viability of *in vivo* plants. *In vivo* plants are usually exposed to environmental stresses that can enhance their resilience. In contrast, *in vitro* cultures are more susceptible to environment and can develop somaclonal variations negatively affecting their viability (Bairu and Kane 2011). While the highest protoplast yield achieved by our protocol is lower than that reported in previous studies (up to 10 million protoplasts per gram; Zhou et al. 2021, Kumar et al. 2023), we have demonstrated that this does not negatively affect successful genome editing experiments.

**Figure 1.**
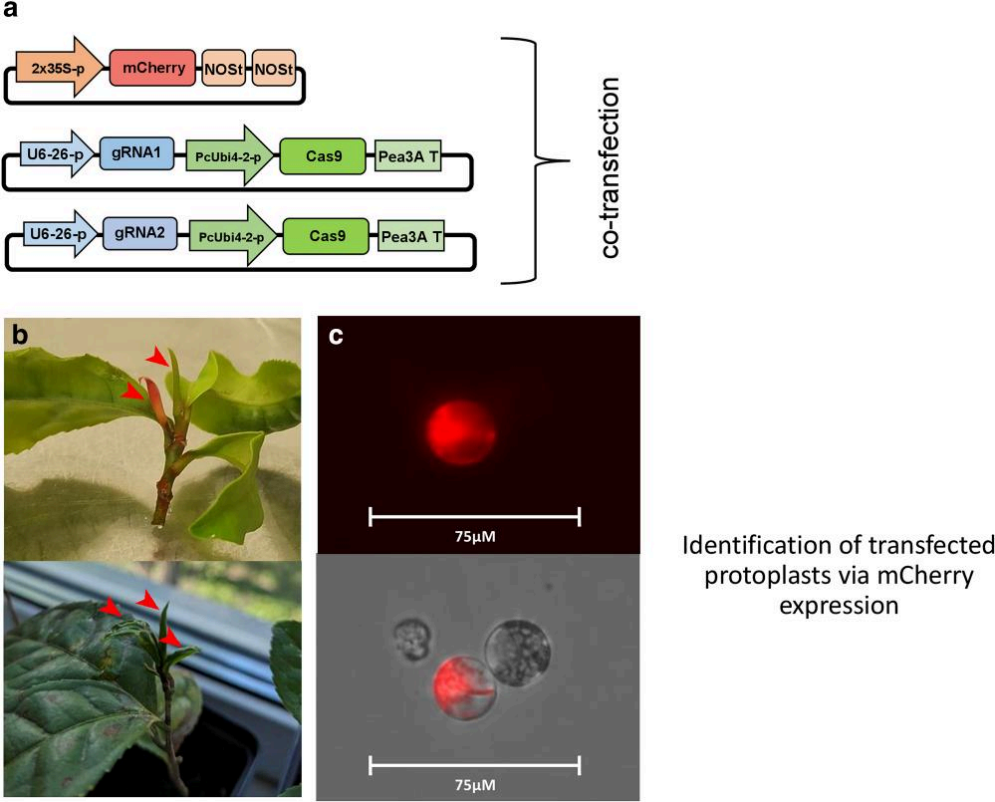
Protoplast isolation and transfection. (a) Vector architectures used for protoplast transfection. mCherry vector architecture used for protoplast transfection: 2 × 35S-p, cauliflower mosaic virus CaMV 35S doubled-enhanced promoter; mCherry, mCherry fluorescent protein gene; NOSt, nopaline synthase terminator. gRNA/Cas9 vector: U6-26-p, *A. thaliana U6-26* promoter; gRNA, chimeric single-guide RNA; PcUbi4-2-p, *Petroselinum crispum Ubiquitin-4-2* promoter; Cas9, plant codon-optimized *cas9* endonuclease gene; Pea3A T, *Pisum sativum* 3A terminator. (b) Young leaves as marked with red arrows were used to isolate protoplasts from both *in vitro* (top photo) and *in vivo* (bottom photo). (c) Identification of transfected protoplasts by expression of the mCherry reporter; red filter (top), overlay of bright field and red filter (bottom).

**Figure 2.**
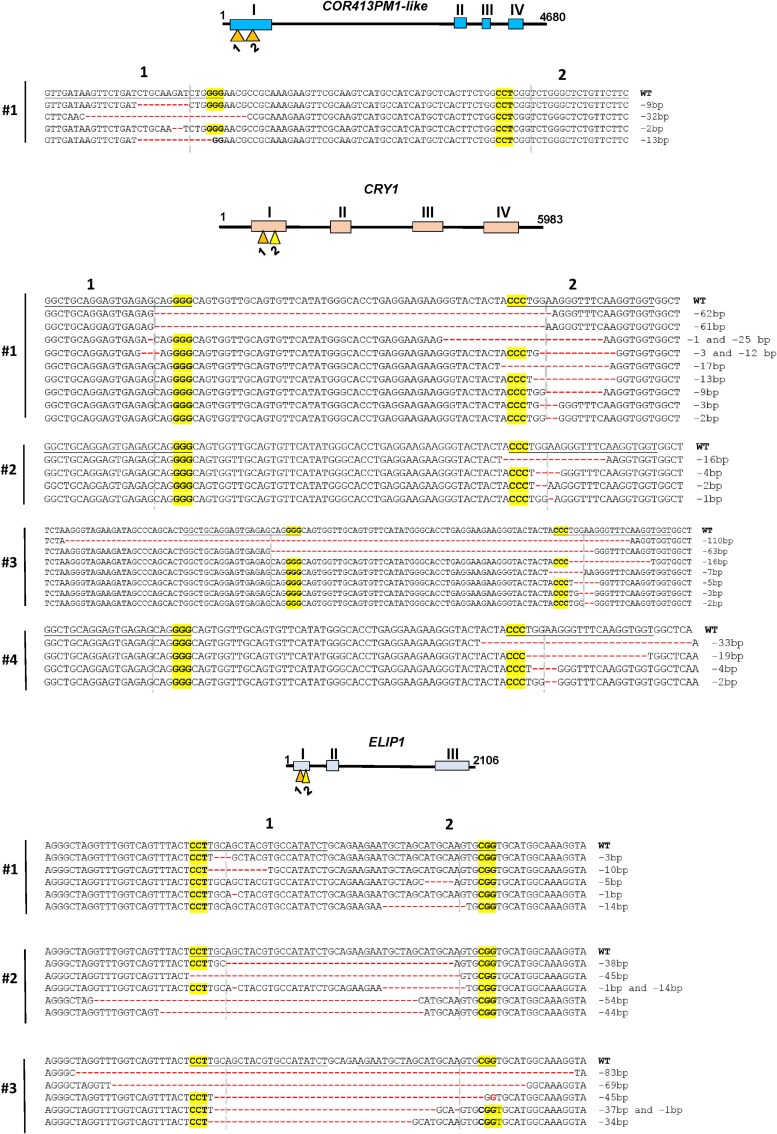
Cas9/gRNA gene editing of the *COR413PM1-like*, *ELIP1* and *CRY1* genes in protoplasts. The gene schemes and the most frequent mutations are indicated. Exons are depicted as rectangles and target motifs as triangles. Replicates are depicted with numbers. Target motifs are underlined, PAMs in bold. The cleavage sites of Cas9 are indicated by dashed lines.

*In vitro* leaves were only used for the *ELIP1* replicate 1 transfection (Fig. 1 and Table 1). Each protoplast sample was co-transfected with three vectors: two gRNA/Cas9 vectors and a reporter vector for transfection efficiency control (see Fig. 1a). Mutations were detected in all three genes and almost every target motif (except of COR-2), with deletions found between two target motifs and small insertions/deletions (indels) found within individual target motifs (Fig. 2). The ELIP-1/ELIP-2 gRNA combination showed the highest combined efficiency (up to 100%), while the COR-1/COR-2 pair showed the lowest efficiencies: 8.4% and 0% per target motif, respectively (Table 1). The mutagenesis efficiency was inconsistent between different replicates; for example, the ELIP-1 and ELIP-2 gRNAs showed very high efficiency in one replicate and zero efficiency in another. The CRY-1 and CRY-2 guide RNAs showed consistent efficiency (24%–31%) across all four replicates.

**Table 1.** The normalized mutation frequency during Cas9/gRNA gene editing in cv. Kolkhida tea protoplasts.

	*COR413PM1-like*				
Replicate	1	2	3	4	control
Transfection efficiency, %	25	20	10	−	−
Reads number	2665	1737	3115	−	3033
Mutation efficiency, %	8.4	0	0		−
Deletions between target motifs	−	−	−	−	−

	*CRY1*				
Replicate	1	2	3	4	control
Transfection efficiency, %	50	25	28	50	−
Reads number	1724	986	1205	980	1997
Mutation efficiency, %	31.6	36	26.4	24	−
Deletions between target motifs	+	−	+	−	

	*ELIP1*				
Replicate	1	2	3	4	control
Transfection efficiency, %	20	30	32	10	−
Reads number	1882	2095	240	1315	2612
Mutation efficiency, %	60	57	100	0	−
Deletions between target motifs	−	+	+	−	−

**Figure 3.**
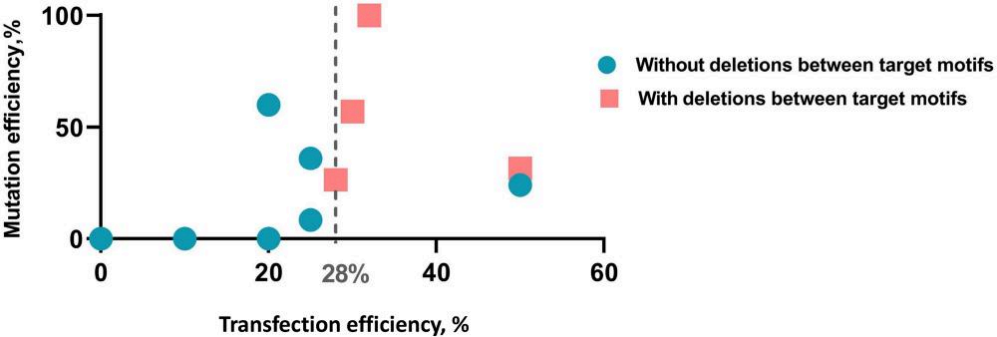
The relationship between transfection efficiency and mutation efficiency in cv. Kolkhida tea protoplasts. Circles indicate samples without deletions between target motifs, and squares indicate samples with deletions.

Protoplast transfection efficiencies of up to 100% can be achieved in model species and genotypes, such as rice or barley. However, in non-model species protoplast transfection efficiencies are often considerably lower, ranging from 7% to 10% (Biswas et al. 2022, Egorova et al. 2024). The transfection efficiency achieved in cv. Kolkhida protoplasts ranged from 10% to 50%. We observed clear dependence of mutation frequency and pattern on protoplast transfection efficiency. Greater transfection efficiency corresponded to the greater mutagenesis efficiency and presence of deletions between target motifs. Mutations in target motifs were only observed in the samples with a >20% transfection efficiency. The deletions between target motifs were observed only in protoplast samples with transfection efficiencies more than 28% (Table 1, Figs 2 and 3). A moderate non-significant positive correlation (Rs = 0.53, *P* = .095) was detected between transfection and mutagenesis efficiencies, and a moderate significant positive correlation (Rs = 0.63, *P* = .036) was detected between transfection efficiency and the presence of deletions between target motifs (Supplementary Figure S4).

Logically, deletions between target motifs can only occur when both vectors have been successfully delivered to the same cell and expressed sufficiently. Studies such as Locatelli et al. (2003) have shown that those protoplasts which were successfully transfected with one plasmid are also highly likely to incorporate co-delivered plasmids. The co-transfection of plant protoplasts with several plasmids is widely used to screen for subcellular localization, promoter activation, and protein interactions (Zhang et al. 2011), as well as for screening gene editing elements (Hernandes-Lopes et al. 2023, Shao et al. 2024). However, the low overall transfection efficiency can limit the effectiveness of co-transfection. In our experiments, deletions between the target motifs were predominantly observed in samples with higher transfection efficiency. This suggests that the robust delivery of the constructs is essential for inducing such complex genomic modifications.

Based on these observations, we recommend that researchers exercise caution when interpreting genome editing results obtained from samples with low transfection efficiency. Transfection rates above 30% may be necessary to reliably detect certain types of mutation, such as deletions, which require the simultaneous activity of multiple editing components. Also, when transfection efficiency is high, co-delivery of separate vectors may be a viable strategy. Conversely, under conditions of low transfection efficiency, assembling all gRNAs and the reporter gene into a single plasmid may be more reliable, ensuring simultaneous expression and editing activity within individual cells.

Testing genetic constructs in protoplasts allows their functionality to be validated and their *in vivo* efficiency to be assessed, thereby addressing the limitations of online tools in accurately predicting gRNA performance. While a gRNA may demonstrate high effectiveness *in silico*, this does not necessarily guarantee efficient *in vivo* gene editing. Out of the six gRNAs selected for knocking out of three target genes, two exhibited almost no efficiency *in vivo*. Similar discrepancies have been reported in *Nicotiana benthamiana*, chickpea, potato, and wheat (Brandt et al. 2020, Naim et al. 2020, Badhan et al. 2021, Egorova et al. 2025), where gRNAs that performed well *in silico* or *in vitro* exhibited low or no editing efficiency *in vivo*. This can be due to chromatin conformation and target motif accessibility which affect the editing efficiency *in vivo*. Remarkably, in our study two gRNAs with low efficiency belonged to the same gene, *COR413PM1-like*, suggesting that chromatin structure may contribute to the reduced editing efficacy.

**Figure 4.**
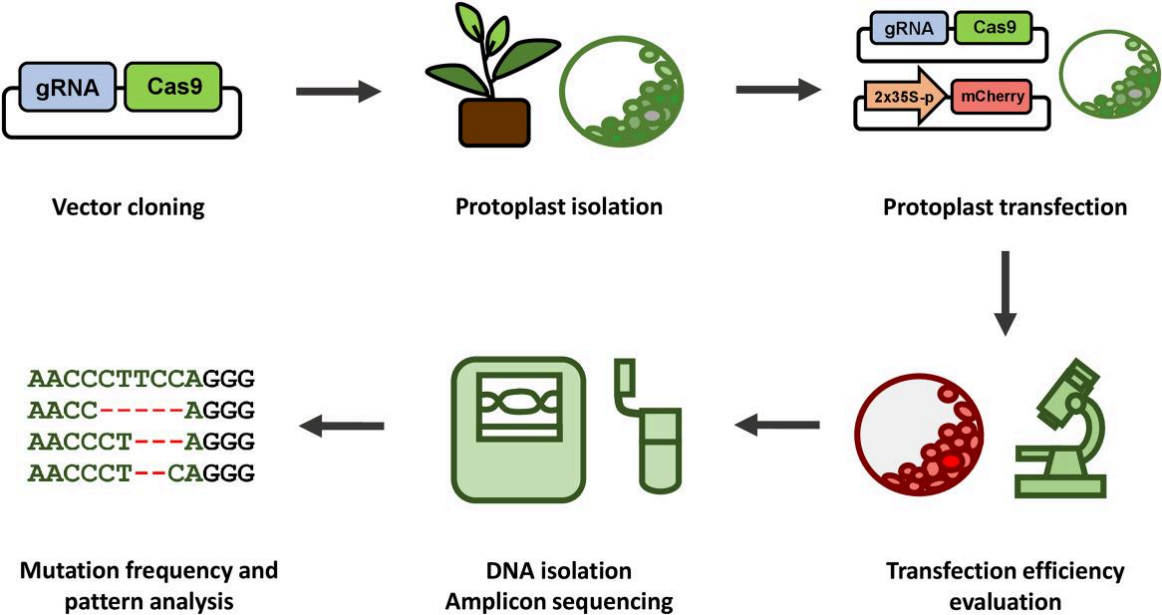
The developed pipeline for tea plant protoplast genome editing.

Developed protocol for tea plant protoplast genome editing (Fig. 4) can serve for various applications. Moving forward, tea protoplasts offer a versatile platform for other molecular screening methodologies. For instance, protoplasts from the related species *Camellia oleifera* were used to investigate specific protein subcellular localization and protein-protein interactions (Lin et al. 2023). Multiplex editing strategies—such as PTG-CRISPR and ribozyme-based systems—have been successfully applied in other species using protoplasts (Xia et al. 2022). Emerging tools such as base and prime editing, as well as CRISPRa/i, can be optimized in protoplasts for precise manipulation of gene activity before moving into stable systems (Gentzel et al. 2020, Westberg et al. 2023, Jeong et al. 2025). In some species, protoplasts are used for regeneration and the production of transgene-free genome-edited plants, which remains particularly challenging for woody species, including tea plant. However, the future application of developmental regulators may help overcome this obstacle (Xu et al. 2024).

The future functional validation of target cold-related genes can be performed in stable transgenic or edited tea lines. Phenotypic assays under controlled cold stress can be conducted on lines with overexpression or knockout of the target genes to assess their functional impact. As protoplast regeneration remains a technical barrier in tea plants, the expression cassettes containing gRNAs and Cas9 obtained in our study can be transferred to binary vectors for *Agrobacterium*-mediated transformation (Mondal et al. 2001, Qianru et al. 2017). In parallel, the heterologous expression of these target genes can be performed in model systems such as *A. thaliana* or *Nicotiana* spp. (Guo et al. 2019).

## Conclusion

One of the main challenges in tea biotechnology remains its recalcitrance to stable genetic transformation. In this context, the use of protoplasts provides a valuable platform for optimizing genetic construct configurations. This study demonstrates that tea protoplasts can be used for RNA-guided Cas9 gene editing experiments, with vectors harbouring standard gRNA and Cas9 expression units designed for dicot plants. Successful co-transfection of plasmids and the combined effect of gRNAs were demonstrated in our study, but only at transfection efficiencies above 28%. We suggest that more complex genome editing systems can also be adapted and optimized for tea plants using the protoplast test platform.

## Supplementary Material

plaf056_Supplementary_Data

## Data Availability

The original contributions presented in the study are included in the article/Supplementary material, further inquiries can be directed to the corresponding author.
