# Supplementary material for: Targeted genome modification in protoplasts of a tea cultivar Kolkhida using RNA-guided Cas9 endonuclease
Source: AoB Plants. 2025 Oct 4;17(5):plaf056. doi: 10.1093/aobpla/plaf056 (PMC12550894; doi:10.1093/aobpla/plaf056)

Supplementary materials

Table S1. gRNAs' characteristics.

**gRNA**	**Target position in the gene**	**Target motif with PAM (bold)**	**Target-specific part of guide-RNA**	**Predictive activity (WuCRISPR)**
COR-1	65-87	gttctgatctgcaagatctg**ggg**	gttctgatctgcaagatctg	96
COR-2	135-157	gaagaacagagcccagaccg**agg**	gaagaacagagcccagaccg	100
ELIP-1	85-107	agatatggcacgtagctgca**agg**	agatatggcacgtagctgca	91
ELIP-2	113-135	agaatgctagcatgcaagtg**cgg**	agaatgctagcatgcaagtg	98
CRY-1	1079-1101	ggctgcaggagtgagagcag**ggg**	ggctgcaggagtgagagcag	100
CRY-2	1151-1173	accaccttgaaacccttcca**ggg**	accaccttgaaacccttcca	96

Table S2. Oligonucleotides, used in this work.

Name	Sequence 5‘-3‘	Amplicon size (bp)	Application
COR-1-F	ATTGGAAGAACAGAGCCCAGACCG		Target-specific parts of gRNA creation
COR-1-R	AAACCGGTCTGGGCTCTGTTCTTC		
COR-2-F	ATTGGTTCTGATCTGCAAGATCTG		
COR-3-R	AAACCAGATCTTGCAGATCAGAAC		
ELIP-1-F	ATTGAGATATGGCACGTAGCTGCA		
ELIP-1-R	AAACTGCAGCTACGTGCCATATCT		
ELIP-2-F	ATTGAGAATGCTAGCATGCAAGTG		
ELIP-2-R	AAACCACTTGCATGCTAGCATTCT		
CRY-1-F	ATTGGGCTGCAGGAGTGAGAGCAG		
CRY-1-R	AAACCTGCTCTCACTCCTGCAGCC		
CRY-2-F	ATTGACCACCTTGAAACCCTTCCA		
CRY-2-R	AAACTGGAAGGGTTTCAAGGTGGT		
COR_F	GTTGATTTCACATAGAGAACATCGG	222	NGS and Sanger sequencing
COR_R	TAGCAGCGATTGAAGCTATCC		
ELIP_F	TCCTGGCAAGACCGGTGA	294	
ELIP_R	CCATTAGGGCACTACGACTACC		
CRY_F	ATTTGGGTTTTGGAAATCTTGGAGG	398	
CRY_R	GGATGATCCAAAGGGGGAATTGA		

Figure S3. Target genes sequence alignments to the references. Target motifs with PAM are indicated.

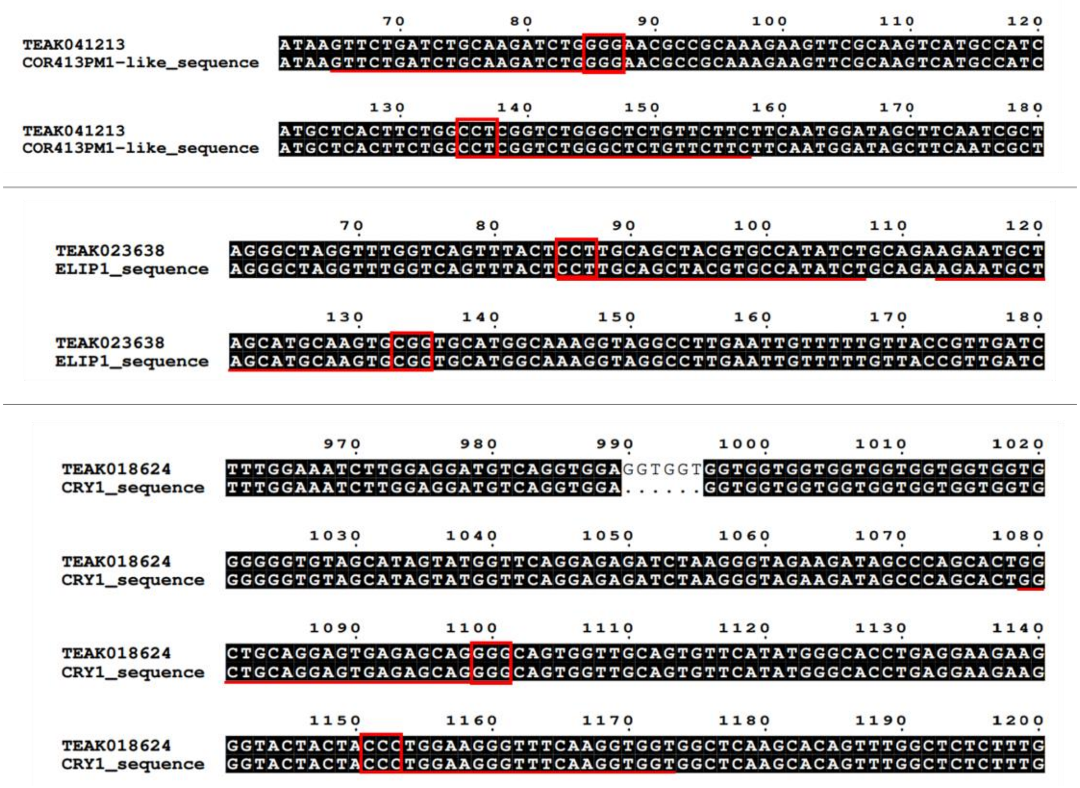

Figure S4. Spearman correlation analysis performed in R, version 4.3.2, between (a) transformation and mutagenesis efficiencies and (b) transformation efficiency and the presence of deletions between targeting motifs.

(a)

```
        Spearman's rank correlation rho

data:  teaprot$tr and teaprot$mut
S = 103.89, p-value = 0.09519
alternative hypothesis: true rho is not equal to 0
sample estimates:
      rho
0.5277778
```

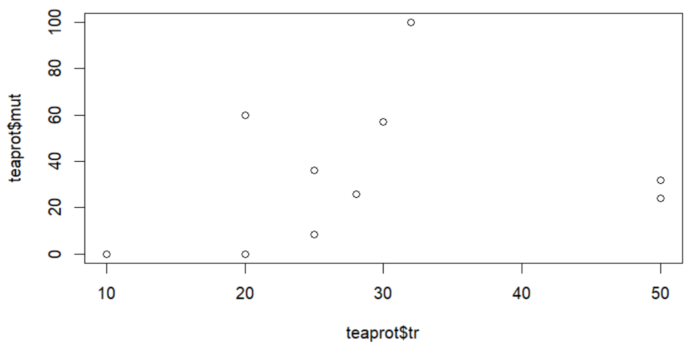

(b)

```
        Spearman's rank correlation rho

data:  teaprot$tr and teaprot$del
S = 80.679, p-value = 0.03647
alternative hypothesis: true rho is not equal to 0
sample estimates:
      rho
0.6332785
```

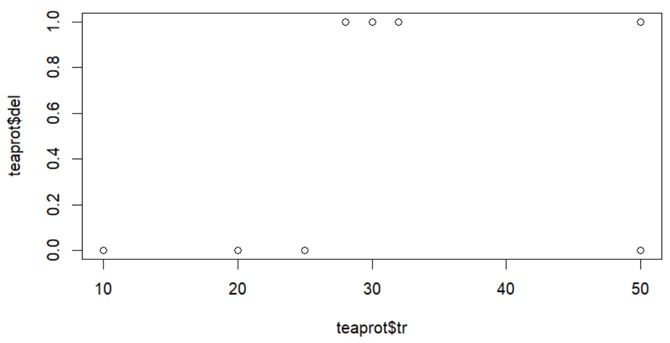

Supplement: plaf056_Supplementary_Data [file plaf056_supplementary_data.pdf]
